# Spousal support during pregnancy in the Nigerian rural context: a mixed methods study

**DOI:** 10.1186/s12884-021-04135-3

**Published:** 2021-11-15

**Authors:** O. Arisukwu, C. O. Igbolekwu, I. A. Oyekola, E. J. Oyeyipo, F. F. Asamu, O. N. Osueke

**Affiliations:** Landmark University, Omu-Aran, Nigeria

**Keywords:** Spousal support, Pregnancy, Rural communities, women’s health and wellbeing, Expected and received supports

## Abstract

**Background:**

Pregnancy constitutes a global health concern, thus the need for spousal support during this period cannot be overemphasized. This study examined the kinds of support pregnant women expected and received from their spouses as well as the effect of such supports during pregnancy, labour, and delivery.

**Methods:**

The study adopted both quantitative and qualitative methods of data collection. The respondents were selected using multistage and simple random sampling techniques.

**Results:**

Findings showed that respondents expected and received maximum support from their spouses during pregnancy, labour, and delivery. Spiritual support such as praying and fasting was top of the kinds of support pregnant women expected and received from their husbands during pregnancy and delivery. Others include helping in house chores, financial provision, taking care of other children, accompanying to labour room, and sexual support. More than three-quarters of the respondents stated that maximum support from their husbands made pregnancy, labour, and delivery easier. Cramer’s V showed that the association between support and husbands’ occupation was 0.233 and Pearson Chi-square showed that the association was statistically significant *χ2*(2) = 27.894,*p* < .001.

**Conclusion:**

The study concluded that spousal support during pregnancy was high among rural women in Southwestern Nigeria, and it impacted positively on their wife’s period of pregnancy, labour, and delivery. A high level of spousal support should be sustained to promote family bonding and development as well as reduce maternal and child mortality.

**Supplementary Information:**

The online version contains supplementary material available at 10.1186/s12884-021-04135-3.

## Background

Pregnancy imposes a great deal of physical, psychological, and emotional pressure on women. Its outcome is often unpredictable; thus, a pregnant woman requires a lot of support from people around her, especially her husband.

Pregnancy is a period of increased vulnerability and both parents are expected to face new challenges during this period [1]. The period of pregnancy could be characterised by several disorders such as depression and anxiety [2]. Death resulting from pregnancy-related conditions, otherwise known as maternal mortality is currently a global health concern.

Reports have it that about 295,000 women died as a result of pregnancy and childbirth issues in the year 2017, and a majority (94%) of these deaths were from low- and middle-income countries which could have been prevented [3]. World Health Organization (2019), report further showed that 86% of maternal deaths were from Sub-Saharan Africa and Southern Asia and that sub-Saharan Africa alone accounted for two-thirds of maternal deaths. Nigeria is currently among the 15 countries considered as ‘very high alert’ or ‘high alert’ with maternal mortality rates (MMR) between the range 31 and 11,580 [3, 4] Nearly 20% of all global maternal deaths occur in Nigeria [5, 6]. Nigeria’s estimated MMR was over 800 per 100,000 in 2015.

It is reported that a Nigerian woman has a 1 in 22-lifetime risk of dying in pregnancy, childbirth, or postpartum/post-abortion compared to 1 in 4900 in developed countries [6, 7].

While pregnancy may not be considered a disease, a lot of expectant mothers in Nigeria die from pregnancy-related complications yearly. At least, one in every thirteen Nigerian woman dies during pregnancy or childbirth, and this is more prevalent in the rural population, where there are fewer health facilities [3, 8]. More so because the decisions on the kind of health facilities to consult even during pregnancy mostly rest on the male counterparts, especially husbands [9, 10] The prevailing patriarchal system in rural areas explains this. Currently, 48.8% of the population of Nigeria is rural (99,027,063 people in 2020), among whom about 45% are women [11]. Considering the predominance of the patriarchal and polygamous systems that characterise rural communities in Nigeria, it is pertinent to examine the experiences of rural women during pregnancy, labour, and delivery.

There is a need for adequate support for a woman during pregnancy, especially from the spouse. Spousal presence may serve as a pain relief for a woman in labour or during childbirth, and spousal support is necessary, in that spouses are preferred companions for women in labour [2, 12]. Some scholars have observed that although fathers desired to be actively involved in the antenatal and intrapartum periods, they are limited by factors such as levels of informational support, attitudes towards involvement, qualities of marital relationship, relationships with their parents, as well as the diverse perceptions about spousal support during pregnancy [13]. Spousal support includes but is not limited to physical, emotional, psychological, spiritual, and financial supports that a male partner gives to a woman during pregnancy, labour, and delivery. It entails support in areas such as house chores, antenatal visits, sex, spiritual and emotional supports.

Previous studies have shown that socio-cultural dynamics may elicit different perceptions about spousal support during pregnancy [14–16]. For example, while a Fulani woman is expected to suppress pains during pregnancy for her to gain entry into the rite of passage [17, 18], a typical Yoruba or Igbo woman is very vocal in the expressions of feelings, especially during pregnancy. Hence the Yoruba and Igbo women may anticipate more spousal support during pregnancy than their Fulani counterparts. Also, a male offspring of a polygamous home may not have a heightened impression about the need to support his wife during pregnancy [13]. Such men may not see reasons why a woman should need special support during pregnancy. He may have the impression that his mother and her co-wives have carried several pregnancies despite little or no support from his father. This may have implications on the health-seeking behaviour of the woman, especially as husbands reserve the economic power in decisions about the healthcare of a wife. This is often prevalent in rural societies where women are predominantly economically dependent on their spouses [9, 10, 19]. A study conducted in Mali confirmed that men are traditionally socialized to be superior in family decision-making as they reserve the duty of providing financial support to the family [20]. Hence, financial support is a masculine way for men to take part in pregnancy. However, assisting the wife in other house chores or attending antenatal services requires that men are ready to enter into the female domain. This may even be frowned at by relatives and friends. Such economic powers are backed by the patriarchal system and cultural norms and practices inherent in most sub-Saharan African communities [19, 20].

Furthermore, some factors such as levels of information, attitudes towards involvement, qualities of marital relationship, relationships with parents, as well as socio-demographic factors like occupation, level of education, age, among others may constitute major predetermining factors for the kinds of support expected and received from husbands during pregnancy, Labour and delivery [3, 20]. These factors could also determine the extent of support a husband gives his wife during these periods. Furthermore [20], also posited that social norms, gendered belief systems, and practical barriers hinder men’s involvement in maternal health. Although a husband’s support during pregnancy is pertinent, it could also be marred by the attitude of the wife during pregnancy. Several women are known to exhibit some forms of aggression during pregnancy and this could pose limitations to their husband’s support or even aggravate physical or emotional abuse by either of the spouses [21]. Thus, it becomes very imperative to understand the kind of support women expect from their spouses during this period. Some women may expect that their spouses be physically present in the labour rooms and during delivery, while others may anticipate more support during the period of pregnancy, especially in the area of domestic chores, sex, spiritual, finance, among others. There are also assumptions that women who are supported by their husbands may have better pregnancy outcomes compared to those who were not. Hence it is important to understand the kind of support a woman expects and what she receives from her spouse during pregnancy, labour, and delivery. Although significant progress may have been made on the need for spousal support during pregnancy [22, 13], there is a dearth of literature on the kinds of support women expect and receive from their spouses during pregnancy, labour, and delivery, particularly among rural communities in Nigeria. This study, therefore, investigated the kind of support women expect and receive from their spouses, among rural women in South-western Nigeria.

Objectives of the study:

The major objective of this study was to examine Spousal Support during Pregnancy in the Nigerian Rural Context. Specifically, the study focused on the following objectives:

1. To examine the kind of support women expected and received from their spouses, during pregnancy in the study area.
2. To investigate the effect of spousal support on pregnancy, labour, and delivery in South-Western Nigeria.

## Method

### Study area

The study was conducted among rural women in Southwestern Nigeria. This Geo-political region in Nigeria is made up of six states namely: Ekiti, Lagos, Ogun, Ondo, Osun, Oyo.

### Study design

The employed mixed-methods descriptive research design.

### Study participants and sampling procedures

A multi-stage sampling method was used to select the study sample. At the first stage, three states (Osun, Ondo, and Ekiti) were purposefully selected from the six states that make up South-western Nigeria based on the low developmental condition of those states [22]. In each of the three senatorial districts in each state, one local government area was selected (making 3 local government areas per state and a total of 9 local government areas for the 3 states understudy). In the third stage of the sampling, one rural community was selected from each of the nine local governments using the purposive sampling technique based on their assessment as meeting the national statistical offices’ definition of the term rural area [23]. Furthermore, in each rural community, 10 compounds were selected purposively based on the availability of women who had experienced or who is experiencing pregnancy. (In rural communities, compounds usually comprise many nuclear families).

A simple random technique was used to select 6 previously or currently pregnant women from each compound, making a total of 540 women selected from across the 90 compounds in the three states of South-western Nigeria.

Also, the purposive sampling technique was used to select respondents for qualitative data. Thus 9 traditional birth attendants (TBA) and 9 trained midwives (one TBA and one trained midwife per rural community) based on their experiences as maternal health workers and that they attended to pregnant women during antenatal and delivery.

### Sample size determination

The sample size of the study was determined using Fisher’s formula:, where n is the minimum sample size; z is the standard normal deviation; p is the prevalent rate or the level of spousal support during pregnancy, labour, and delivery since the rate is uncertain in Nigeria due to insufficient data from the previous study, this study adopts 50% as recommended in the literature; q is 1-p, and d is the degree of freedom. That is, representing the minimum sample size for this study. Hence, a sample size of 540 respondents was selected for this study after due consideration for the possibility of incomplete responses, the nature of the sampling technique for the study, and the peculiarity of study locations. Consequently, a sample size of approximately 540 respondents (which consist of women who had experienced pregnancy before or who were experiencing pregnancy as at the time the study was conducted) was selected.

### Variable measurements

The major variable in the study “Spousal Support” was measured in two dimensions: support during pregnancy and labour and delivery. Expected supports during pregnancy were measured using the following variables; house chores, taking care of other children, praying, and fasting, among others. Support during labour and delivery was measured using variables such as; massaging the wife, feeding the wife, comforting, holding of hands, sitting beside the wife, showing her understanding during the pregnancy and delivery, spiritual support, financial support, among others. Related and vital questions were asked on these variables and responses were obtained and presented on tables.

### Data collection

The method of data collection was triangulated using both qualitative and quantitative techniques. For the quantitative study, the questionnaire was the major research instrument used to elicit data from women aged 17 and above, who have been pregnant before or are currently pregnant. The questionnaire was structured. It comprised both open and close-ended questions designed to measure the objectives of the study. It was subdivided into subsections to reflect the specific objectives of the study. The questionnaire after due validation was also transcribed into Yoruba, which is the local language of the respondents.

Also, a key informant interview guide was used to collect qualitative data from traditional birth attendants and trained midwives. The interviews were conducted by trained research assistants, who were closely supervised by the authors who anchored the data collection section of the study. The interviews were audiotaped and lasted between 30 to 50 min for each interviewee. It was conducted in the local language (Yoruba) and later transcribed in English.

Traditional Birth attendants/ Midwives:

Based on their experiences as maternal health workers, these respondents provided first-hand information about husband’s supports for pregnant women, especially during antenatal, labour and delivery. They witness and conduct delivery sections for pregnant women and thus could tell how supportive a spouse could be during labour and delivery. TBA was selected because many women in the rural areas studied still patronised them, however, a good number of the pregnant women also patronize the modern health facilities, this also necessitated our choice of trained midwives.

The data collection process spanned for a period of 3 months. The researchers employed the services of research assistants, who were purposefully trained for the collection of both quantitative and qualitative data.

### Data analysis

Data from the quantitative study were analysed using descriptive statistics such as frequencies and percentages, simple and stacked column bar charts, cross-tabulation, correlation, and chi-square. Also, qualitative data were analysed using content analysis.

Considering that the study was triangulated, the qualitative data was used to support the findings of the quantitative data. Hence qualitative and quantitative results were used to complement each other, hence there were no divergent results.

### Ethical review and approval

Ethical approval was sought and obtained from Landmark University Ethical Review Board. All the principles governing human research were observed. Respondents were briefed about the study and its expected outcomes and benefits. They were assured of the confidentiality and anonymity of their responses, their consent to be part of the sample population was also obtained.

## Result

### Description of the study sample

Results showed that all the respondents (or women) have husbands. While the mean, median, and standard deviation of the respondents’ ages were 34 years, 33 years, and 8.7, those of the husbands were 39 years, 38 years, and 8.9 respectively. The average ages of wives and husbands at first pregnancy were 27 years and 32 years, and the standard deviations of their ages at first pregnancy were 4.4 and 4.5 respectively. The mean and standard deviation of respondents’ years of marriage were 8 years and 7.5 respectively. The number of pregnancies and of children differed as the means were 3 and 2 children respectively with a standard deviation of 1.6. The majority of the respondents and their husbands had tertiary education and were Yoruba. While a majority of both the respondents (57.8%) and their husbands (51.5%) were public servants, a minority (1.9%) of the respondents were not employed and none of their husbands was unemployed. A detailed description of the study sample is provided in Table 1.

**Table 1:** Description of study sample

	Wives		Husbands	
	N	%	n	%
**Age (**mean, median, mode, min., range, SD)	33.9, 33, 28, 17, 57, 8.7		38.6, 38, 35, 22, 58, 8.9	
**Age as at First Pregnancy**	26.9, 26, 25, 16, 23, 4.4		31.6, 32, 32, 21, 24, 4.5	
**Years of marriage**	8.3, 6, 1, 1, 49, 7.5		Same	
**Number of pregnancy ever had**	2.5, 2, 2, 0, 8, 1.6		–	
**Present number of children**	2, 2, 2, 0, 8, 1.6		–	
**Number of children outside wedlock**	–		0.6, 0, 0, 0, 9, 1.4	
**Education**				
No formal education	8	1.6	24	4.7
Primary education	14	2.7	10	1.9
Secondary education	105	20.3	65	12.6
Tertiary education	389	75.4	417	80.8
**Ethnicity**				
Yoruba	411	79.6	451	87.4
Igbo	71	13.8	40	7.8
Hausa	34	6.6	25	4.8
**Occupation**				
Unemployed	10	1.9	0	0
Self-employed	135	26.2	167	32.4
Public servant	298	57.8	266	51.5
Private employees	73	14.1	83	16.1
**Overall**	**516**	**100**	**Same**	

Source: Researcher’s field study 2020

### Husbands received and expected support during pregnancy/ effects of supports during pregnancy, labour, and delivery

Investigation on husbands’ supports during pregnancy showed that expected support and received support differed among the respondents, although not significantly (see Table 2). While 93.2% of the respondents received support from their husbands during pregnancy, a little higher percentage (95.2%) of them expected support from their husbands. Findings on reasons for expecting support from husbands showed that enhancement of mothers’ health (46.3%) was the major reason for expecting support from husbands. Also, 23.6% of the respondents stated that they expect support from their husbands because ‘it is necessary for easy pregnancy and delivery. Other minor reasons for expecting support from husbands were; ‘is the husbands’ responsibility’ (7.8% is morally and spiritually good’ (6.8%), ‘due to mothers’ weakness during pregnancy (6.8 percent), and ‘because the pregnancy is for both party’ (2.9 percent) and ‘being the first child’ (1.0%). The few respondents (4.8%) that didn’t expect support from their husbands also gave their reasons and they stated that it was because their ‘husbands do not care or count it’ or because their ‘husbands were always busy’.

**Table 2:** Husbands received and expected support during pregnancy and its Effects on pregnancy, labour and delivery (*N* = 516)

	N	%
**Husbands’ support during pregnancy (received)**		
Yes	481	93.2
No	35	6.8
**Husbands’ support during pregnancy (expected)**		
Yes	491	95.2
No	25	4.8
**Reasons for expecting husbands’ support**		
It enhances mothers’ health	239	46.3
It is morally and spiritually good	35	6.8
It is necessary for easy pregnancy and delivery	122	23.6
Being the first child	5	1.0
Due to mothers’ weakness during pregnancy	35	6.8
It is the husbands’ responsibility	40	7.8
Because the pregnancy is for both party	15	2.9
Husband does not care or count it	18	3.5
Husband is always busy	7	1.3
Effect of minimum and maximum supports during pregnancy, labour and delivery		
Pregnancy, labour and delivery were very easy during maximum support	392	76.0
Pregnancy, labour and delivery were difficult despite maximum support	84	16.3
Pregnancy, labour and delivery were easy despite minimal support	30	5.8
Pregnancy, labour and delivery were very difficult during minimal support	10	1.9

Source: Researcher’s field study 2020

The above findings were also corroborated by a traditional birth attendant (TBA) in Osun State who also posited that many of the women that were delivered from child birth in their hospitals were supported by their husband’s presence and the point of labour and delivery. The respondent had this to say;

*Although a few of the husbands accompany their wives during antenatal visits, the majority of them are often present during labour and delivery to support their wives (TBA/KII/ Osun /2020)*.

This shows that a husband’s presence during child labour and delivery was perceived by even the TBAs as being supportive to pregnant women.

Similarly, the Midwifes also posited that the spouses of the respondents supported them during pregnancy, labour and delivery. Another respondent also said:

*Many of the husbands are meeting up to our expectations these days. Oftentimes, we prefer to have them around their wives during labour and delivery as it also helps to facilitate decision-making processes especially during emergencies (Midwife/KII/Ondo/2020).*

These responses from both quantitative and qualitative data imply that a husband’s presence at the delivery sections will facilitate quick decision making especially in cases of emergency decision making such as cesarean sections. Hence, the need to examine the impact of these supports.

### On the effect of minimum and maximum supports during pregnancy, labour and delivery

The study further investigated the effect of lowest and highest support on pregnancy, labour, and delivery. More than three-quarters (76.0%) of the respondents stated that maximum support makes pregnancy, labour, and delivery easier. Also while 16.3% of the respondents affirmed that pregnancy, labour, and delivery were difficult despite maximum support, 5.8% of the respondents claimed that pregnancy, labour, and delivery were easy despite minimum support. Lastly, the results showed that pregnancy, labour, and delivery were very difficult because of minimal support.

### Support providers during pregnancy

**Fig. 1:**
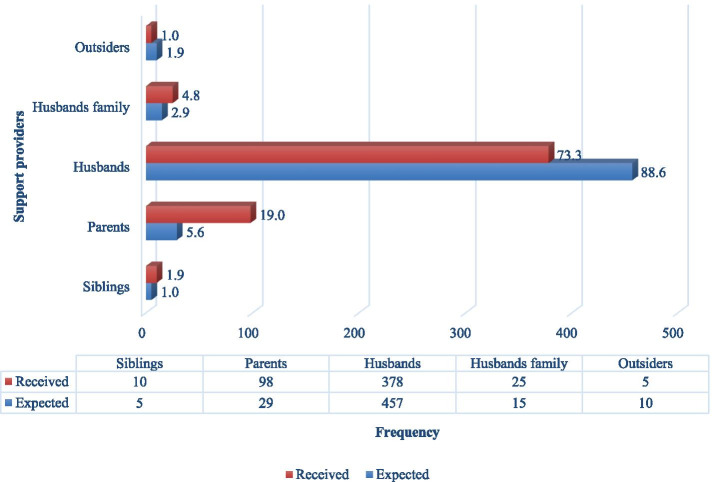
Support providers during pregnancy (*N* = 516). (Source: Researchers field work 2020)

Findings presented in Fig. 1 showed that for received support, husbands provided the highest support (73.3%), while outsiders (1.0%) constituted the least received support. Husbands were equally the highest expected support providers (88.6%), while siblings were the least expected support providers (1.0%).

### Kinds of support expected and received from husbands during pregnancy

Since husbands were found to be the highest support providers during pregnancy, the study further examined the kinds of support expected and received from husbands during pregnancy. Table 3 showed that the first kind of support respondents expected from their husbands was spiritual support such as praying and fasting with 55.0% of the respondents supporting this. This was followed by respondents’ expectations of their husbands to support them in the areas of house chores (46.1%), financial provision (39.7%), taking care of other children (34.7%), accompanying to labour room (19.4%), sexual support (18.4%), accompanying to antenatal (16.3%), and accompanying to the delivery room (13.4%). The findings showed that the rating of the kinds of support respondents expected from their husbands was the same as the kinds of support they received from their husbands up to the fourth rating with spiritual support, house chores support, financial support, and taking care of other children topping the list. Only the fifth (sexual support) and sixth (accompanying to labour room) kinds of support received were interchanged with what was expected.

**Table 3:** Kinds of support expected and received from husbands during pregnancy *N* = 516)

	Expected		Rank	Received		Rank
	n	%		n	%	
House chores	238	46.1	2nd	260	50.4	2nd
Taking care of other children	179	34.7	4th	243	47.1	4th
Praying and fasting	284	55.0	1st	287	55.6	1st
Accompany to antenatal	84	16.3	7th	83	16.1	7th
Accompany to labour room	100	19.4	5th	102	19.8	6th
Accompany to delivery room	69	13.4	8th	78	15.1	8th
Financial provision	205	39.7	3rd	252	48.8	3rd
Sexual support	95	18.4	6th	110	21.3	5th
Other areas such as going to the market, assisting in official work, etc	10	1.9	9th	5	1.0	9th

(Source: Researchers field work 2020)

These findings from qualitative data were also corroborated by a midwife in Ekiti State who emphasized the importance of husband’s support during pregnancy. Specifically, she enlisted praying and fasting, financial support among others, as very vital support systems for the pregnant woman. The respondent said:

*The husband's support for a pregnant woman is very vital. At least in house chores, finances, and most importantly praying and fasting for their wives during this period will greatly assist the woman (midwife/KII/Ekiti/2020)*

Corroborating this, another respondent posited that:

*Prayer is very important for every pregnant woman. So the husbands support their wives with prayers (Midwife/KII/Osun/2020).*

This implies that prayer and fasting by a spouse was rated high and appreciated by both the respondents and the midwives during pregnancy and delivery. It could also mean that the awareness of their husband’s support also places these pregnant women in the right psychological mind frame to deliver their babies.

### Expected support during delivery and labour

What kinds of support do women expect from their husbands during labour and delivery? This study found that during labour, 46.1% of the respondents expected spiritual support from their husbands, while 3.9% expected their spouse to feed them during labour. However, this order above was not followed during delivery indicating a change in their expectation. For instance, during delivery, while the respondents still considered spiritual support to be number one on the list, sitting beside them, financial support, comfort, understanding, and holding their hands were considered second, third, fourth, fifth, and seventh expected support during delivery respectively. Further details are presented in Table 4.

**Table 4:** Expected support during delivery and labour

	During labour		Rank	During delivery		Rank
	n	%		N	%	
Massage	83	16.1	5th	–	–	–
Feeding	20	3.9	9th	–	–	–
Comfort	119	23.1	3rd	99	19.2	4th
Holding	73	14.1	6th	34	6.6	7th
Sitting beside	54	10.5	7th	139	26.9	2nd
Understanding	154	29.8	2nd	94	18.2	5th
Spiritual	238	46.1	1st	278	53.9	1st
Financial	118	22.9	4th	129	25.0	3rd
Others such as chatting, gaming, etc	35	6.8	8th	35	6.8	6th

(Source: Researchers field work 2020)

Findings from the qualitative study confirmed this. For example, a midwife from Osun state confirmed that most husbands are always present during their wife’s deliver. She states that:

*‘Most husbands always stay around here praying for their wives especially during the labour and delivery period. Their wives enjoy their support when they are around (Midwife/KII/Osun/2020)*

Also, a traditional birth attendant (TBA) from Ondo State also confirmed that the husbands’ presence during the delivery of his wife was one of her requirements for delivery procedure. She stated as follows:

*‘I usually insist that the pregnant women come with their husbands especially on the day of delivery, at least that way they (husbands) will also support them through prayers and encouragement (TBA/KII/Ondo/2020)*

Some midwives also support the presence of husbands in the delivery room so that they can serve as an encouragement to their wives during delivery.

*“I like the husbands to come into the delivery rooms to witness the delivery”(Midwife/KII/Ondo/2020).*

These findings imply that husband support during pregnancy, labour and deliver is pertinent as it serves as a source of encouragement for pregnant women.

### Rating of husbands’ level of support

**Fig. 2:**
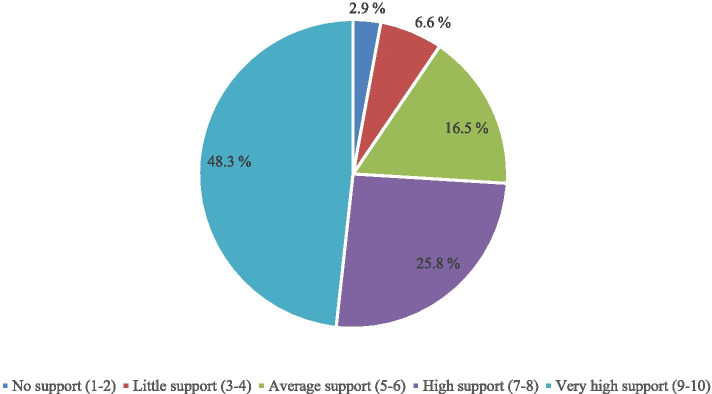
Rating of husbands’ level of support. (Source: Researchers field work 2020)

Further investigation was carried out on respondents’ rating of their husbands’ level of support and the results showed that respondents rated their husbands very high in terms of the level of support they received from them. In other words, almost half (48.3%) of the respondents rated their husbands’ level of support very high (on a scale of 9 to 10). This was followed by 25.8% of the respondents who rated their husband’s level of support to be high (on a scale of between 7 and 8). Other respondents rated their husbands’ level of support to be average (16.5%), little (6.6%), and none (2.9%). Figure 2 presents the rating of husbands’ level of support graphically.

One of the midwives who were interviewed in Ekiti State posited that there is an increase in the rate of husband’s support during pregnancy in their community. She said,

*“Spousal support during pregnancy has improved significantly in our rural communities. On a scale of 1 to 10, I will give the husbands 8 (Midwife/KII/Ekiti 2020)*

This implies that most men are beginning to embrace the need to support their wife’s during pregnancy and this development may have implications on reducing maternal and infant mortality in Nigeria.

### Spousal status

On Table 5, this study further found that the majority (77.7%) of the respondents were the only spouse of their husbands while the husbands of 22.3% of the respondents had one or more wives aside from them. In addition, the husbands of 19.4% of the respondents had only one wife outside wedlock while the husbands of 1.0% each of the respondents had more than one wife.

**Table 5:** Spousal status

	N	%
**Spousal status**		
My husband has only me as his wife	401	77.7
My husband has other wife/wives	115	22.3
**Number of other wife/wives**		
None	401	77.7
One wife	100	19.4
Two wives	5	1.0
Three wives	5	1.0
Above three wives	5	1.0

(Source: Researchers field work 2020)

An interviewee in Osun State also confirmed the notion that having only one wife will enhance support during pregnancy. The respondent had this to say;

*‘Most men prefer to marry only one wife these days. A man with only one wife gives her undivided support during pregnancy (TBA/KII/Osun/2020)’.*

This implies that the number of wives a husband has will also determine the level of his support to them during pregnancy, labour, and delivery.

### Association between husbands’ support (received) and some selected variables

Findings from the association between husbands’ support and spousal status on Table 6 showed that among husbands who provided support for their wives, a majority (78.2%) had only one wife while others (21.8%) had more than one wife. The *Phi* statistics showed that the correlation between husbands’ support and spousal status was 0.041, indicating a very weak association [24] and the Pearson Chi-square showed that the correlation was not statistically significant *χ2*(1) = 0.856, *p >* .05. Results from the association between husbands’ support and husbands’ age categories showed that among those who provided support for their wives, the majority (45.5%) were between ages 31 and 40 years, and 29.9% were between ages 41 and 50 years. Only 17.5% of those who provided support to their wives were between ages 21 and 30 years. Although the association between husbands’ support and husbands’ age categories was very weak – Cramer’sV = 0.174 [24] Pearson Chi-square indicated that the association was statistically significant *χ2*(5) = 15.574, *p* < .01. The findings further showed that among those who provided support to their wives, a majority (80.4%) had tertiary education followed by those who had secondary education (12.5%). The association between husbands’ support and husbands’ education was very weak – Cramer’s V = 0.072 [24] and not statistically significant *χ2*(3) = 2.671, *p >* .05. Finally, findings on the association between husbands’ support and husbands’ occupation showed that among those who provided support to their wives, the majority (54.3%) were public servants, while others were self-employed (29.5%) and private sector employees (16.2%). Cramer’s V showed that the association between husbands’ support and husbands’ occupation was 0.233 indicating a weak positive association [24] and Pearson Chi-square showed that the association was statistically significant *χ2*(2) = 27.894,*p* < .001.

**Table 6:** Association between husbands’ support (received) and some selected variables

		Husband support (received)		
		Yes n(%)	No n(%)	
Spousal status	One wife	376 (78.2)	25 (71.4)	*χ*^*2*^ = .856 *Phi* = .041 df = 1, *p* = .355
	More than one wife	105 (21.8)	10 (28.6)	
Husbands’ age	21–30 years	84 (17.5)	15 (42.9)	χ^2^ = 15.574 Cramer’s V = .174 df = 5*, p* = .008
	31–40 years	219 (45.5)	10 (28.6)	
	41–50 years	144 (29.9)	10 (28.6)	
	51–60 years	25 (5.2)	0 (0.0)	
	61–70 years	5 (1.1)	0 (0.0)	
	71–80 years	4 (0.8)	0 (0.0)	
Husbands’ education	No formal education	24 (5.0)	0 (0.0)	χ^2^ = 2.671 Cramer’s V = .072 df = 3, *p* = .445
	Primary education	10 (2.1)	0 (0.0)	
	Secondary education	60 (12.5)	5 (14.3)	
	Tertiary education	387 (80.4)	30 (85.7)	
Husbands’ occupation	Self-employed	142 (29.5)	25 (71.4)	χ^2^ = 27.894 Cramer’s V = .233 df = 2, *p* = .000
	Public servant	261 (54.3)	5 (14.3)	
	Private employees	78 (16.2)	5 (14.3)	
**Total**		**481 (100.0)**	**35 (100.0)**	

(Source: Researchers field work 2020)

## Discussion

This study shows that spousal support during pregnancy, labour, and delivery among rural women in Southwestern Nigeria was high. While the major providers of this support were their husbands, the women expected and received more spiritual supports from their spouses. This support impacted positively on their pregnancies as it made labour and delivery easier.

Women’s expectation of spousal support during pregnancy was slightly different from received support, although both expected and received spousal support were very high in the study locations despite the predominance of a patriarchal system. This contradicts existing findings that socio-cultural dynamics may elicit different negative perceptions about spousal support during pregnancy [13, 16, 20]. Particularly, the welcoming of a new child in the study area comes with a lot of excitement and ceremonies, like naming ceremony, child dedication, among others; thus both partners are grossly involved.

The husbands in the rural communities in South-western Nigeria have proven that irrespective of the power bestowed on them by their patriarchal heritage [10, 24], there is still a need to support their wives especially during pregnancy. This could also be because of the close family bond in the rural areas. Most husbands reside permanently with their wives and are mostly engaged in jobs that do not entail leaving separate from their wives compared to what is obtainable in the urban areas.

There were many reasons for pregnant women to be expecting support from their spouses during pregnancy and some of which were that such support enhances mothers’ health, it eases pregnancy, labour, and delivery periods, and that it is the husbands’ responsibility to support their wives, among others. When women expect and receive support from their husbands, it makes them happy and this, in turn, enhances their wellbeing which has a capacity of improving the overall mental health of both the mother and the baby since happiness results from the experiences of individuals as well as their satisfaction in life including their family. Receiving support from husbands during pregnancy, therefore, has a positive effect on women and baby’s overall wellbeing, and such always makes pregnancy, labour, and delivery easier. This makes spousal support a vital requirement for pregnant women’s mental health and wellbeing. Existing studies have also shown that spousal support is necessary in that husbands are preferred labour companions for women in labour, and that spousal presence makes delivery less painful but more life-fulfilling [2, 12].

Among all the support providers during pregnancy, husbands were the ones that the respondents expected the most support from. This underscores the importance of husband support during pregnancy, labour, and delivery [12, 25].

On the kinds of support pregnant women expected from their spouses both during pregnancy and at delivery, the study observed that the first kind of support the respondents expected from their husbands was spiritual support such as praying and fasting. Others include house chores or emotional support, financial provision, taking care of children (if any), accompanying them to antenatal, labour and delivery room, sexual support, massage, holding, sitting beside, and feeding during labour, among others. Although existing studies found financial support to be most paramount among the support pregnant women expected and received from their spouses, the findings of this study contradict this pre-existing notion. Similarly, financial support was also considered a masculine way for men to take part in pregnancy [20], however, this study establishes that spiritual and emotional supports are more pertinent and better appreciated by women during the period of pregnancy than financial support. Although the importance of financial support cannot be overlooked, it should be complemented by spiritual, emotional, and physical supports.

Husbands’ level of support during pregnancy was rated very high. This shows that spousal support during pregnancy is significantly high among the rural women of Southwestern Nigeria despite the prevailing patriarchal cultural practice, which imposes male dominance over females. Furthermore, the study also found that majority of the respondents were from nuclear families with only one wife. The nuclear family system may be a good reason for increased spousal support among the studied group. This is consistent with the findings of an existing study on the impact of polygamy on spousal support. Therefor it may be necessary to study the level of spousal support during pregnancy among women in urban areas as well as in polygamous family settings.

The study further revealed that among those who provided support to their wives, the majority had tertiary education followed by those who had secondary education. Similarly, findings on the association between husbands’ support and husbands’ occupation also showed that among those who provided support to their wives, the majority were public servants, while others were self-employed and private sector employees. Finding from this study also validates the assertions by [13] that demographic variables such as age, education, occupation, number of wives married among others; affect the level of spousal support during pregnancy [14].

### Recommendations

The study recommends that a high level of spousal support should be sustained to promote family bonding and development. Since the study was limited to spousal support among married women in the rural areas, we recommends that further studies be done on the impact of spousal support among women in urban areas, as well as polygamous families from other ethnic groups in Nigeria.

## Conclusion

The study concludes that spousal support is very important and appreciated by women during pregnancy, labour, and delivery. Spiritual support was found to be the major support expected and received by women in the study area. Spousal support was admitted by the respondents as having a positive impact on them during pregnancy, labour, and delivery. Hence spousal support during pregnancy was high among rural women in South-western Nigeria, and it impacted positively on their wife’s period of pregnancy, labour, and delivery.

## Supplementary Information

**Additional file 1.** BMC questionnaire for spousal support.

**Additional file 2.**

## Data Availability

All dataset generated and/or analysed during the current study are available from the corresponding author on reasonable request.
