# Supplementary material for: Spousal support during pregnancy in the Nigerian rural context: a mixed methods study
Source: BMC Pregnancy Childbirth. 2021 Nov 15;21:772. doi: 10.1186/s12884-021-04135-3 (PMC8591893; doi:10.1186/s12884-021-04135-3)
Supplement: Supplementary file 1 — Additional file 1. BMC questionnaire for spousal support. [file 12884_2021_4135_MOESM1_ESM.docx]

Spousal Support during Pregnancy in the Nigerian Rural Context

Dear Respondent,

This questionnaire is designed to assist the researcher in a study on Spousal Support during Pregnancy in the Nigerian Rural Context. We solicit your honest response to the questions herein. Be assured that all information provided will be treated with utmost confidentiality as none will be traced to you. You are also free to decline response to any question and can also withdraw from the exercise at any time you deem necessary.

Thank you.

**Consent statement**: Tick the one that is appropriate

I voluntarily agree to participate as a respondent and also give my consent for publication of this study ( )

I am not willing to participate in this study and do not give my consent for publication of this study. ( )

**Instruction: Kindly underline your response to the questions or provide written answers were applicable**

**Section A. Respondent’s socio demographic data**

1. What is your age as at last birthday?……………………………………………………
2. What is your highest Educational qualification? ..............................................................
3. Which is your ethnicity? .....................................................................…..........................
4. What is your occupational category?……………………………………………………
5. What is your monthly income? .........................................................................................
6. What was your age as at first pregnancy…………………………………………………
7. How long have you been married?……………………………………………………….
8. How many pregnancies have you had? .....................................................................
9. How many children do you have now?……………………………………………………
10. What is your religion?..............................................................................................

**Section B. Husband socio demographic data**

1. What is your husband’s age as at last birthday?……………………………………………
2. What is your husband’s highest Educational qualification?..................................................
3. Which is your husband’sethnicity?....................................................................…...............
4. What is your husband’s occupation? …………………………………………
5. What is yourhusband’s monthly income? ............................................................................
6. What was your husband’sage as at your first pregnancy…………………………………
7. How many other children does your husbandhave ?…………………………………

**Section C:**

1. Do you agree that husbands should support their wives during pregnancy? a. Yes b. No
2. If yes or no what are the reasons for your answer……………………………………………………………

………………………………………………………………………………………………………………

1. Do you think your husband’s support has any effect on your pregnancy outcome? a. Yes b. No
2. If yes or no give reasons for your answer. ………………………………………………………………… ……………………………………………………………………………………………………………
3. Does your husband’s support encourage you to have?
4. Fewer children b. Many Children
5. Does your husband’s lack of support encourage you to have?
6. Fewer children b. Many Children
7. Are you usually aggressive to your husband during pregnancy?

Yes b. No

1. If yes or no give reasons for your answer…………………………………………………………………….

……………………………………………………………………………………………………………..

1. What influences your aggression?
2. Lack of finance
3. My husband beats me
4. Lack of love and care from my husband
5. Lack of understanding from my husband
6. The pregnancy condition
7. Other specify..............................................................................................................

**Section D.**

1. When am pregnant, I expect maximum support from my a. siblings b. parentsc. husband d. friends e. husband’s familyf. outsiders
2. When am pregnant, I receive maximum support from my a. siblings b. parents c. husband d. friends e. husband’s family f. outsiders
3. In what areas did your husband support you during pregnancy?
4. Help with house chores
5. Taking care of our other children
6. Praying and fasting for you.
7. Accompany you to antenatal
8. Accompany you into the labour room
9. Accompany you into the delivery room
10. Financial support
11. Sexual support
12. Other specify...........................................................................................................
13. In what areas would you want your husband to support you during pregnancy?
14. Help with house chores
15. Taking care of our other children
16. Praying and fasting for you.
17. Accompany you to antenatal
18. Accompany you into the labour room
19. Accompany you into the delivery room
20. Financial support
21. Sexual support
22. Other specify...........................................................................................................
23. Has your husband ever supported you during any of your pregnancy a. Yes b. No
24. Which of the pregnancy did you receive maximum support a. First b. Second c. Thirdd.Fourth. e. Fifth f. others please specify……………
25. In your view, whichreason mostly influences his support during pregnancy?
26. Medical condition of the baby
27. Your medical condition
28. Gender of child,
29. Position of child (i.e1^st^, 2^nd^ 3^rd^ child)
30. Presence of a family member around
31. No specific reason
32. Other Specify....................................................................................................................
33. Which of the pregnancy did you receive minimum support a. First b. Second c. Third d.Fourth. e. Fifth f. others please specify………
34. In your view, what reasonmostly accounts for his lack of support?
35. Loss of his job
36. Gender of child
37. Position of child (i.e1^st^, 2^nd^ 3^rd^ child)
38. Presence of a family member
39. Medical condition of the baby
40. Your medical condition
41. Paternity issues
42. No specific reason
43. Other, specify................................................................................................................................................
44. What kind of support do you expect from your husband during labour? .......….………………………………………………………………………………………………..........
45. What kind of support do you expect from your husband during delivery? ………………………………………………………………………………………………………………
46. Please tick if there were incidence of the following during pregnancy?a. verbal abuse, b. quarrel. c. fight d. violence during the pregnancy e. Other, specify................................
47. On a scale of 1-10 please rate your husband’s level of support to you during pregnancy?
48. 1-2 No support. b. 3- 4 little support c. 5-6 average support d. 7-8 high support e. 9 -10 very high support
49. Are you the only spouse to your husband?

Yes b. No

1. How many other wives aside you are married to your husband?
2. None
3. One
4. Two
5. Three
6. Four
7. Other, specify..........
8. Do you think this has implication (s) on his level of support to you during pregnancy?
9. Yes b. No
10. If yes or no state your reason for the answer? ............................................................................. ............................. **.**..................................................................................................................

**Section E.**

1. The support you receive from your husband,
2. Makes the period of pregnancy easy
3. Makes the labour easy
4. Makes delivery easy
5. Helps reduce the stress and pains
6. Gives you psychological and emotional relief especially during complications.
7. Has no effect on the pregnancy
8. The lack of support you receive from your husband,
9. Makes the period of pregnancy difficult
10. Makes the labour difficult
11. Makes delivery difficult
12. Increased the stress and pains
13. Added to the psychological and emotional trauma thereby leadingtocomplications.
14. Has no effect on the pregnancy
15. Compare your pregnancy outcome during minimal and maximum support from your husband.
16. The Pregnancy, labour and delivery was easier during maximum support
17. The Pregnancy, labour and delivery was difficult despite maximum support
18. The Pregnancy, labour and delivery was easy despite minimal support
19. The Pregnancy, labour and delivery was very difficult because of minimal support
