# Supplementary material for: Spousal support during pregnancy in the Nigerian rural context: a mixed methods study
Source: BMC Pregnancy Childbirth. 2021 Nov 15;21:772. doi: 10.1186/s12884-021-04135-3 (PMC8591893; doi:10.1186/s12884-021-04135-3)
Supplement: Supplementary file 2 — Additional file 2. [file 12884_2021_4135_MOESM2_ESM.pdf]

Landmark University
PMB 1001, Omu-Aran, Kwara State
www.lmu.edu.ng

Chancellor: DR. DAVID OYEDEPO (FNAE)
Vice-Chancellor: PROFESSOR ADENIYI OLAYANJU
Registrar: OYINLOYE, A. FOLA (MNIM)

CENTRE FOR RESEARCH, INNOVATION
AND DISCOVERIES (LUCRID)

Director: Prof. B. O. Adebesin
Email: dlucrid@lmu.edu.ng

26th May 2021

Editor-in-Chief,
BMC pregnancy and Child Journal

COMPLIANCE WITH ETHICAL STANDARDS

I write to attest and approve that Dr. Chisaa, IGBOLEKWU (of the Department of Sociology, Landmark University) et al., complied with all ethical standards involving the use of human subjects in her research work titled "Spousal Support during Pregnancy in Nigerian Rural Context". Statements or testimonies of the participants will remain confidential and shall only be used for research purposes.

Kind regards.

Professor. B.O. Adebsin
Director, LUCRID
